# Safety and therapeutic effects of personalized transcranial direct current stimulation based on electrical field simulation for prolonged disorders of consciousness: study protocol for a multi-center, double-blind, randomized controlled trial

**DOI:** 10.3389/fneur.2023.1184998

**Published:** 2023-06-29

**Authors:** Mi-Jeong Yoon, Hyun Mi Oh, TaeYeong Kim, Soo-Jin Choi, Woo Hee Choi, Hong Soo Jung, Sung Chul Lim, Yeun Jie Yoo, Hye Jung Park, Bo Young Hong, Geun-Young Park, Donghyeon Kim, Tae-Woo Kim, Sun Im, Seong Hoon Lim

**Affiliations:** ^1^Department of Rehabilitation Medicine, St. Vincent’s Hospital, College of Medicine, The Catholic University of Korea, Seoul, Republic of Korea; ^2^Department of Rehabilitation Medicine, National Traffic Injury Rehabilitation Hospital, Gyeongki-do, Republic of Korea; ^3^Research Institute, NEUROPHET Inc, Seoul, Republic of Korea; ^4^Department of Rehabilitation Medicine, Bucheon St. Mary's Hospital, College of Medicine, The Catholic University of Korea, Seoul, Republic of Korea; ^5^Division of Nuclear Medicine, Department of Radiology, St. Vincent's Hospital, College of Medicine, The Catholic University of Korea, Seoul, Republic of Korea; ^6^Department of Anesthesiology and Pain Medicine, St. Vincent's Hospital, College of Medicine, The Catholic University of Korea, Seoul, Republic of Korea; ^7^Department of Neurology, St. Vincent's Hospital, College of Medicine, The Catholic University of Korea, Seoul, Republic of Korea; ^8^Department of Rehabilitation Medicine, Seoul St. Mary’s Hospital, College of Medicine, The Catholic University of Korea, Seoul, Republic of Korea

**Keywords:** non-invasive brain stimulation, DOC, consciousness, transcranial direct current stimulation, neuromodulation, clinical trial, minimal consciousness state, vegetative state

## Abstract

**Background:**

Disorders of consciousness (DOC) resulting from acquired brain injury (ABI) increase the mortality rate of patients, complicate rehabilitation, and increase the physical and economic burden that DOC imposes on patients and their families. Thus, treatment to promote early awakening from DOC is vital. Transcranial direct current stimulation (tDCS) has shown great potential for promoting neuro-electrochemical activity. However, previous tDCS studies did not consider structural damage or head and brain lesions, so the applicability of the results to all DOC patients was limited. In this study, to establish a patient-specific tDCS treatment plan considering the brain lesions of and damage sustained by DOC patients, we considered the electric field calculated by a the “finite electric” three-dimensional brain model based on magnetic resonance images. This protocol was developed to aid tDCS treatment of actual patients, and to verify its safety and effectiveness.

**Methods/design:**

Twenty-four patients with DOC after ABI will be enrolled in this cross-over trial. All participants will receive typical rehabilitation combined with sham tDCS and typical rehabilitation plus personalized tDCS (P-tDCS). Each interventional period will last 2 weeks (30 min/day, 5 days/week). The primary outcome [score on the Korean version of the Coma Recovery Scale-Revised (K-CRS-R)] will be assessed at baseline and the end of the first day of the intervention. Secondary outcomes (K-CRS-R at 1 week and 2 weeks after experimental session and quantitative EEG changes quantitative electroencephalography changes) will be measured at baseline and the end of week 4. Adverse events will be recorded during each treatment session.

**Discussion:**

For patients with neurological disorders, tDCS has served as a painless, non-invasive, easily applied, and effective therapy for several decades, and there is some evidence that it can improve the level of consciousness of patients with DOC. However, variability in the effects on consciousness among subjects have been reported and personalized strategies are lacking. This protocol is for a randomized controlled trial designed to validate the effectiveness and safety of P-tDCS combined with typical rehabilitation for DOC.

**Clinical trial registration:**

https://cris.nih.go.kr, identifier KCT0007157.

## Introduction

Acquired brain injury can result in prolonged disorders of consciousness (DOC) including coma, “unresponsive wake syndrome” (UWS; also called vegetative state [*VS*]) (1) and a minimally conscious state (MCS) (2). Several studies have attempted to determine the effectiveness of brain stimulation techniques, such as deep brain stimulation (DBS) (3), transcranial magnetic stimulation (4), and transcranial direct stimulation (tDCS), for improving the level of consciousness of patients with DOC (5). In particular, tDCS therapy is emerging as a non-invasive treatment, with no side effects such as seizures (6).

tDCS is a form of cortical stimulation in which anode and cathode electrodes are attached to the scalp or forehead and continuous direct current is applied. Several studies have reported that anodal stimulation of the damaged cortical area in DOC patients improves the function of the stimulated area. The dorsolateral prefrontal cortex (DLPFC) is the most important target region to improve consciousness in DOC patients. One study showed that a single session of tDCS over the left DLPFC improved the level of consciousness in 43% of patients in an MCS (7) In a study of UWS and MCS patients, tDCS was used to activate the left DLPFC and restore consciousness, and all MCS patients showed immediate clinical improvement after the tDCS intervention. Patients who received a second tDCS treatment 3 months after the first showed additional clinical improvement and the emergence of consciousness (8). In another study, consciousness was restored in chronic MCS patients through repeated tDCS treatment, and a significant change in the Coma Recovery Scale-Revised (CRS-R) score was seen compared with a sham tDCS treatment. Moreover, recovery of consciousness was maintained for up to 1 week after the end of tDCS treatment (9). tDCS has also been applied in clinical settings, as it can easily be customized by varying the position, size, number, and current of electrodes without any major adverse events.

Several studies have attempted to improve the effectiveness of tDCS for patients with DOC. The retrospective study described above divided patients with DOC into groups that did and did not recover consciousness (9). In that study, left DLPFC tDCS correlated with less metabolic impairment in distant brain regions, as well as in regions presumably stimulated by tDCS. Studies have begun to explore why tDCS treatment is not effective in all patients; differences in the severity of the disability, location or size of the brain lesion, and structural characteristics of the brain around the lesion have all been implicated.

tDCS has emerged as a major research interest. However, studies have only been performed retrospectively; no actual patients have been recruited and personalized tDCS (P-tDCS) has not been performed. Precise modeling and simulation are often precluded by the patient’s surgical history, a pre-existing implant device, or a skull defect. Datta (10) performed a simulation study to test the effect of a pre-existing device on the tDCS-induced electrical field and reported no significant interference. We will apply P-tDCS to patients with DOC, with a focus on safety. The goal is to provide evidence that tDCS can be applied as a new treatment method other than medication or existing physical therapy for DOC patients who have been excluded from several previous studies, considering that it may not be safe to receive tDCS treatment due to skull defects or medical history. To achieve that goal, this study will consider the electric field values generated in the target area for consciousness recovery with a simulation-based P-tDCS method and perform a tDCS simulation. This clinical trial aims to develop P-tDCS programs to restore consciousness in DOC patients in a *VS*/UWS or MCS. Sham tDCS will serve as the control. Whether P-tDCS treatment based on brain magnetic resonance imaging (MRI)-optimized tDCS is safer and more effective than sham tDCS will be assessed.

## Materials and methods

### Trial design

**Figure 1:**
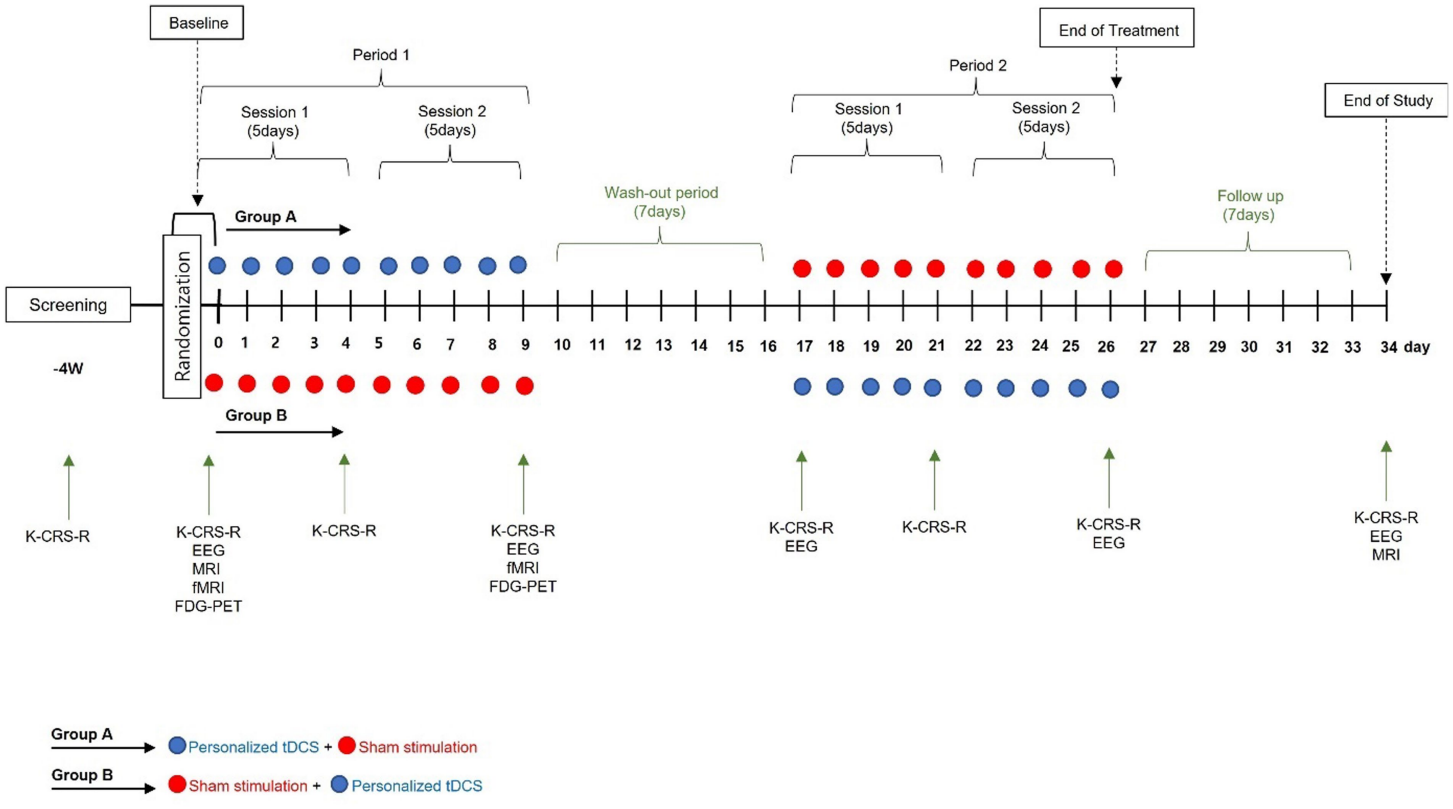
The study protocol.

A prospective, randomized placebo-controlled cross-over double-blind multicenter phase 2 feasibility study will be performed (Figure 1; Table 1). P-tDCS will be compared to sham tDCS in patients in a *VS*/UWS or MCS (8, 11). Typical rehabilitation will also be applied, such as physical or occupational therapy for 1–2 h per day (5 days per week). The therapies will be performed passively, i.e., not in a goal-oriented manner, due to the debilitating nature of DOC.

**Table 1:** Study design.

	Screening	Baseline	Period 1		Outcome	Wash-out period	Period 2		End of study
			Session 1	Session 2			Session 1	Session 2	
Visit	V1	V2	V3 ~ V6	V7 ~ V11	V11	2 weeks	V12 ~ V16	V17 ~ V21	V22
ENROLLMENT									
Informed consent	**V**								
Eligibility screen	**V**	**V**							
Randomized allocation		**V**							
Taking a clinical photo	**V**								
MRI-based simulation and planning for tDCS		**V**							
INTERVENTIONS									
Personalized tDCS Sham-tDCS		**V**	**V**	**V**			**V**	**V**	
ASSESSMENTS									
Outcome variables		**V**							
Vital signs	**V**	**V**	**V**	**V**	**V**	**V**	**V**	**V**	**V**
Physical examination	**V**	**V**							
K-CRS-R		**V**			**V**				**V**
EEG		**V**							**V**
NCS-R		**V**	**V**	**V**	**V**	**V**	**V**	**V**	**V**
MRI, fMRI, and PET scan		**V**							**V**
Other variables Adverse events		**V**	**V**	**V**		**V**	**V**	**V**	**V**

### Participants involvement and ethics approval

This protocol was approved by the Korean Ministry of Food and Drug Safety and complied with the ethical standards of the Declaration of Helsinki. The final protocol was approved by the Ethics Review Boards of St. Vincent’s Hospital and Bucheon St. Mary’s Hospital (Catholic University of Korea; approval number: XC21DDDS0158), as well as the National Traffic Injury Rehabilitation Hospital (approval number: NTRH-21027). Written informed consent will be obtained from each participant’s legal guardian.

### Recruitment

Participants will be screened and recruited from three hospital in the Republic of Korea: St. Vincent’s Hospital, Bucheon St. Mary’s Hospital, and the National Traffic Injury Rehabilitation Hospital.

### Inclusion and exclusion criteria

The inclusion criteria that will be applied in the study are as follows: aged 19–80 years; acquired brain injury patients diagnosed with *VS*/UWS or MCS based on the results of the Korean version of the Coma Recovery Scale-Revised (K-CRS-R), administered at least twice within 1 week (12); stable, without any change in medication or other treatments for underlying diseases at least 1 week before the screening date, and scheduled to receive medication or treatment during the study period.

The exclusion criteria that will be applied are as follows: alcohol and drug induced change of consciousness; brain tumor; degenerative disease such as Parkinson’s syndrome; unable to undergo tDCS due to scalp disease; a pre-existing implant device in the brain or skull with a location corresponding to the tDCS electrode attachment sites; use of a stimulation device similar to the medical devices used in this study within the last 1 year or experience participating in related clinical trials; clinically unstable vital signs; unsuitable for tDCS due to surgery that caused structural changes in the brain (e.g., lobectomy or extensive cranial defects), and a medical condition that may affect consciousness.

### Enrollment and randomization

Participants will be assigned a random number at baseline and randomized to either Group A or Group B. As this trial uses a cross-over design, the only difference between the two groups is the order of the intervention [Group A: P-tDCS (period 1), washout period (> 2 weeks), and sham tDCS (period 2); Group B: sham tDCS (period 1), washout period, P-tDCS (period 2)].

All participants will be randomly assigned to the groups at a 1:1 ratio according to the order of registration by the investigator. The statistician for this clinical trial will use the latest version of SAS statistical software (SAS Institute, Cary, NC, USA) to issue random numbers. A stratified block randomization method will be used with a predefined block size; institution will be a stratification factor. Envelopes containing the study numbers will be provided to the “investigational device manager” (IDM) before the participants are registered.

To maintain blinding of the IDM, an unblinded investigator will be assigned to each institution, and will provide the participants with devices according to their group assignments. The unblinded investigators will not participate further in the study.

This parallel randomized controlled trial uses a cross-over design given the clinical needs of the DOC patients. There is no gold standard treatment for DOC, although tDCS may be useful. However, use of tDCS is prohibited in Korea, except in clinical trials, and the treatment options for DOC patients are limited. Benefits of tDCS have been reported in some cases of DOC (13). Because sham group patients would not receive any of the benefits of tDCS due to the parallel study design, a cross-over design is used so that all participants will have the same opportunity to receive tDCS.

### tDCS treatment, simulation, and blinding

After being assigned a study number, the IDM will take photographs with a digital camera to determine the condition of the participant’s scalp and forehead; redness or burns caused by the medical device will be checked, and changes will be recorded. This process will help minimize side effects. After unblinding, the IDM will select electrode positions based on simulations performed using Neurophet tES Lab (ver. 3.0; Neurophet, Seoul, South Korea); the electrodes will then be attached. P-tDCS will be performed using Neurophet tES Lab; three-dimensional (3D) T1 MRI images of the participants will be imported into the software, brain tissue will be segmented, and a mesh will be generated. Brain tissue will be divided into eight layers: skin, skull, cerebral and cerebellar white matter, gray matter, cerebrospinal fluid, and affected tissue (14). A 3D brain model will be created based on the segmentation and mesh. Points in front of both ears, the nasion, and the inion will serve as landmarks in the brain model, which will be optimized using the software. To optimize the P-tDCS, 5 × 5 cm^2^ electrodes will be deployed in a representative area of the left DLPFC, which is the target area for stimulation to recover consciousness. The simulation parameters for determining the optimal electrode position include the initial positions of the anode electrode (F3) and cathode electrode (Fp2), which are based on the international 10–20 electroencephalography (EEG) system. The simulation will begin after inputting these parameters. After completing the simulation, the IDM will check the results and positions of the electrodes, and prepare for the actual tDCS intervention.

#### tDCS device

The tDCS treatment will be applied using a battery-driven, portable tDCS device (Neurophet innk; Neurophet) and two sponge-coated 5 × 5 cm^2^ electrodes. The stimulation parameters will be set using the bundled software. The parameters in Group A will be as follows: tDCS mode, current intensity of 2 mA, and simulation time of 30 min. In Group B (sham tDCS), the sham mode of the software will be used; the stimulation time will be the same as in Group A, while the current will be increased to 2 mA over the first 30 s and then decreased to 0 mA over the next 30 s (15). The current will then be maintained at 0 mA for the next 28 min, increased to 2 mA over 30 s, and finally decreased to 0 mA over the next 30 s.

The tDCS device can check impedance in real time. If the impedance is >13 kOhm, the stimulation will be stopped and the IDM will check the condition of the patient’s skin to prevent adverse events.

Each tDCS intervention will be performed 10 times over 2 weeks. Then, the other tDCS intervention will be performed depending on the group assignment. Any rehabilitation programs in which the participants are enrolled can be continued during the study. The electrode locations will be the same in the sham tDCS and P-tDCS groups.

#### P-tDCS

The P-tDCS process consisted of four steps: MRI segmentation, 3D brain modeling, personalized tDCS planning based on the simulation of E-field, and the treatment. The whole process is going to be conducted using NEUROPHET tES LAB. All participants undergo baseline MRI scans before enrollment. On MRI scans, if a skull bone defect (such as a burr hole) is detected in patients with a history of surgery, or if an implant such as a cable or coils is found, the principal investigator will discuss it with a neurology and neurosurgery specialist to decide whether the patient can participate in the study. After confirming that, as the first step for planning tDCS, the MR image segmentation will be semi-automatically labeled into eight layers: skin, skull, cerebral and cerebellar white matter, gray matter, cerebrospinal fluid, and affected tissue (14). If a patient has a pre-existing implant, their MRI will be segmented manually based on computed tomography scans and X-rays. The second process is brain modeling. Based on the labeled images and segmented data, a 3D model of tetrahedron meshes is generated. Third, P-tDCS planning is based on the simulation of the E-field. The tDCS-induced E-field in the 3D head and brain model is computationally simulated based on the finite element method (FEM). For computational simulation, the electrical conductivity was assigned to a head and brain tissue; scalp = 0.465, skull = 0.01 CSF = 1.65, ventricle = 1.65, white matter = 0.126, gray matter = 0.276, affected tissue = 0.8087, all in S/m (16) To plan P-tDCS in NEUROPHET tES LAB, the Left DLPFC is localized according to a widely used method, in which F3 on the 10–20 electroencephalogram (EEG) system is selected as the anode placement site (center of anode electrode over F3). The method for the P-tDCS plan is a built-in tES LAB feature to determine the optimized position of an anode electrode among several candidate positions around F3, based on the E-field of the Lt DLPFC region.

### Outcomes

K-CRS-R: The CRS is a neurobehavioral assessment instrument used to evaluate the state of consciousness of patients with severe brain injury; it is able to predict the treatment outcome with high accuracy. The CRS-R was released in 2004 and reflects the diagnostic criteria for MCS developed by the Aspen Workgroup in 2002 (2); it is the most effective tool for assessing long-term DOC patients and is recommended by the American Congress of Rehabilitation Medicine (17). The CRS-R distinguishes MCS from *VS* based on six sub-domains (auditory, visual, motor, oral movement/language, communication, and arousal). Total scores range from 0 to 23; higher scores indicate a higher level of function. The validity of the K-CRS-R was established through comparison with the CRS-R (18).

EEG: EEG is a reliable, non-invasive modality to examine the state of consciousness of patients with DOC (18). The spectral power, complexity, and functional connectivity of the theta and alpha bands are related to the state of consciousness, and combining behavioral measures with and EEG is optimal for evaluating the possibility of improving a patient’s consciousness. Brain function changes after tDCS will be evaluated in this study *via* power spectral analysis of the brain region of interest. Interactions and connectivity will be evaluated based on the correlations of EEG phase and amplitude (19, 20).

Functional magnetic resonance imaging (fMRI): fMRI can reveal blood flow changes in response to brain activation by various stimuli. An increase in activity in the brain region associated with mental imagery in a patient with a consciousness disorder imagining performing a specific task indicates that the task instructions are being followed. Because it will be difficult to perform fMRI as a routine examination due to the characteristics of the patients in this clinical trial, it will only be used where available as an exploratory analysis.

Fluorodeoxyglucose-positron emission tomography (FDG-PET): PET measures the activity in a brain area within a short period of time. Hypometabolism is particularly severe in the bilateral frontoparietal cortex of patients with long-term unconsciousness, and metabolic recovery in this area is correlated with the recovery of consciousness (21). In this clinical trial, FDG-PET will be conducted only when it is judged as feasible by the investigator and is thus considered as an exploratory endpoint.

Nociception Coma Scale-Revised (NCS-R): The NCS is used to detect pain in patients with impaired consciousness (22, 23). It consists of four subscales that evaluate facial expressions and motor, verbal, and visual responses to noxious stimuli; total scores range from 0 to 12 points. The NCS-R only evaluates motor, verbal, and facial responses, and total scores thus range from 0 to 9 points (24).

#### Primary and secondary outcomes

The primary outcome will be the change in total K-CRS-R score 2 weeks after the baseline assessment. The secondary outcomes will be changes in the total K-CRS-R score after 1 and 2 weeks, and at the end of the study. Score changes in the auditory, visual, motor, oral motor/linguistic function, communication, and arousal domains will be assessed, along with changes in EEG activity. The exploratory endpoints are fMRI and FDG-PET changes after 2 weeks. The safety endpoints are the NCS-R score, vital signs, and concomitant drug use.

### Sample size estimates

The purpose of this study is to validate the feasibility of P-tDCS for consciousness recovery in patients with PDOC. The effect size f of P-tDCS is expected to be at least 0.25 and the power 0.80, so this study should enroll 22 participants, assuming a drop-out rate of 10%, a total of 24 participants will be enrolled, which is G-Power (version 3.1.9.7) software was used to calculate. The sample size is larger than a previous study of MCS patients (9) and is considered as an appropriate basis for pivotal trial design (25).

### Statistical analyses

As stated above, the primary outcome is the change in total K-CRS-R score 2 weeks after the baseline assessment. Repeated-measures analysis of covariance (ANCOVA) will be performed to compare the groups, with the baseline K-CRS-R score included as a covariate. The first secondary outcome to be evaluated will be the change in total K-CRS-R score 1 week after the baseline assessment and at the end of the study. Repeated-measures ANCOVA will be performed to compare the groups, with the baseline K-CRS-R score included as a covariate. The second secondary outcome will be the changes in K-CRS-R subscale scores at 1 week, 2 weeks, and the end of the study compared to the baseline. Repeated-measures ANCOVA will be performed to compare the groups, with the baseline K-CRS-R score included as a covariate. The third secondary outcome will be the changes in EEG 2 weeks after the baseline assessment and at the end of the study. Repeated measures ANCOVA will be performed, with the baseline EEG results included as a covariate. Finally, repeated-measures analysis of variance will be performed to compare the groups at each time point in terms of changes in fMRI and FDG-PET results relative to baseline. Descriptive statistics (mean ± standard deviation and median and range) will be generated for each outcome. A *p*-value <0.05 will be considered significant.

## Discussion

This randomized, multicenter clinical trial will investigate the immediate and delayed effects of tDCS on the level of consciousness and EEG activity of patients with prolonged DOC. Although previous studies have reported improvements in the level of consciousness after applying tDCS to the F3 region of *VS*/UWS and MCS patients, no clear conclusions were drawn due to methodological limitations and differences in effects among studies and individuals. In addition, most previous studies excluded patients with a history of brain surgery. This trial is being performed to overcome these limitations; it is expected to have relatively high internal validity because participants will be randomly assigned to groups, randomization concealment will be implemented, and raters, participants, and the IDM will be blinded.

Although the neurophysiological and electrophysiological effects of tDCS have been confirmed, and safety has been demonstrated (19, 26), its clinical utility remains to be verified. This clinical trial will aim to determine the effects of P-tDCS treatment in UWS and MCS patients, and the role of simulated electric fields. If tDCS can be proven to provide clinical benefits, it could serve as an important treatment for patients with cognitive impairments.

In closing, this trial has been designed to validate the safety and effectiveness of P-tDCS developed based on each participant’s T1-weighted MRI scans and the results of simulations. The aim is to determine whether P-tDCS is a viable therapy for DOC patients with a surgical history, skull defects, or pre-existing implantable device. Recently developed simulation technologies and Neurophet tES LAB software will be used to this end; in particular, the latter will be used for segmentation and 3D brain modeling.

### Trial status

Recruitment of participants started in May 2022 and will be completed in December 2023. This manuscript reports protocol version 2.1 (December 2, 2022).

## Ethics statement

This study protocol was approved by the Korean Ministry of Food and Drug Safety and complies with ethical standard based on the Declaration of Helsinki. Case report forms will be stored where only researchers can access them, and electronic documents are stored with secure, limited access. Data transmission will be encrypted and information that identifies individuals will be removed. The authors plan to disseminate the results in peer reviewed journals and related scientific conferences. The study protocol was approved and reviewed by Institutional Review Board of Catholic University, College of Medicine, St. Vincent’s Hospital, Bucheon St. Mary’s hospital (approval number: XC21DDDS0158). The study protocol was also approved and reviewed by Institutional Review Board of National Traffic Injury Rehabilitation Hospital (approval number. NTRH-21027). The patients/participants’ legal guardians provided their written informed consent to participate in this study.

## Author contributions

M-JY, HO, TK, and S-JC: making concept, making protocol, and writing draft. WC, HJ, SuL, YY, HP, BH, and G-YP: making protocol. DK, T-WK, SI, and SeL: making concept, writing draft, review and finalize of draft. TK and DK: funding. All authors contributed to the article and approved the submitted version.

## Funding

This work was supported by the Promotion of Innovative Business for Regulation-Free Special Zones funded by the Ministry of SMEs and Startups (MSS, Korea) (P0020624).

## Conflict of interest

Neurophet Inc. provided the tDCS equipment used for investigational use. DK has equity in Neurophet, Inc. TK is employed by Neurophet, Inc.

The remaining authors declare that the research was conducted in the absence of any commercial or financial relationships that could be construed as a potential conflict of interest.

## Publisher’s note

All claims expressed in this article are solely those of the authors and do not necessarily represent those of their affiliated organizations, or those of the publisher, the editors and the reviewers. Any product that may be evaluated in this article, or claim that may be made by its manufacturer, is not guaranteed or endorsed by the publisher.
